# Effect of Grafting Rootstock on the Antioxidant Capacity and Content of Heirloom Tomatoes (*Solanum lycopersicum* L.) in Hydroponic Culture

**DOI:** 10.3390/plants10050965

**Published:** 2021-05-12

**Authors:** Jamie Greathouse, Shelby Henning, Mette Soendergaard

**Affiliations:** 1 Department of Chemistry, Western Illinois University, Macomb, IL 61455, USA; jr-greathouse@wiu.edu; 2 School of Agriculture, Western Illinois University, Macomb, IL 61455, USA; s-henning@wiu.edu

**Keywords:** heirloom tomatoes, grafting, antioxidants, lycopene, phenolic content, hydroponic cultivation

## Abstract

Heirloom tomato varieties are in demand by consumers due to high antioxidant levels. However, these varieties are difficult to produce and are prone to disease. To overcome these problems, heirloom tomatoes may be cultivated in hydroponic systems and grafted onto disease-resistant rootstocks. However, it is unknown if the antioxidant content and capacity are affected by grafting. In this study, heirloom (Black Krim and Green Zebra) and standard (Big Beef) varieties were grafted onto wild type (WT) or productive rootstocks (Arnold and Supernatural). The tomatoes were harvested at maturity, freeze-dried, and ground into a powder. Lycopene was extracted using hexane, and the content was determined spectrophotometrically at 503 nm. The antioxidant capacity of methanol extracts was evaluated by the 2,2′-azino-di[3-ethylbenzthiazoline sulfonsyr]sulphonic acid (ABTS) and 2,2-diphenyl-1-picrylhydrazyl (DPPH) assays, whereas the phenolic content was determined using the Folin–Ciocalteu assay. Interestingly, the grafting of Big Beef and Green Zebra onto Supernatural rootstock resulted in an increased antioxidant capacity, as determined by the DPPH assay. Moreover, the phenolic content was changed for Big Beef grafted onto Arnold, and Big Beef and Green Zebra grafted onto Supernatural. Taken together, these results indicate that certain combinations of standard and heirloom tomato varieties and productive rootstocks may influence the antioxidant capacity and phenolic content. These results may be used to guide producers when choosing rootstocks for cultivating hydroponic tomatoes.

## 1. Introduction

In recent years, heirloom tomatoes (*Solanum lycopersicum* L.) have gained popularity in the U.S. market, as more consumers turn to local, organic, and authentic food experiences [1]. Furthermore, consumers are buying more “superfoods,” which are perceived to have health benefits [2,3,4]. Especially, foods with a high content of antioxidants are receiving increased attention due to the well-established relationship between these compounds and the reduced risk of cancer and cardiovascular diseases [5,6,7,8,9]. Antioxidants prevent disease by scavenging free radicals such as reactive oxygen species (ROS) [10,11,12]. Mitochondrial respiration leads to the creation of endogenous radicals, such as the hydroxyl radical (OH•) and superoxide anion (O_2−_•), which oxidize biomolecules, leading to cellular damage and DNA instability [13,14,15,16]. Exogenous radicals often stem from exposure to ultraviolet (UV) radiation, as well as oxidizing agents from pollution and cigarette smoke [11,17,18,19].

Fruits and vegetables with high levels of pigmentation may exhibit increased health benefits due to the antioxidant capabilities of color compounds [20,21]. Tomatoes are highly pigmented and contain many types of antioxidants, including flavonoids, hydroxycinnamic acids, and lycopene [22,23,24,25]. Lycopene is a carotenoid responsible for the red color of tomatoes [8,26]. Heirloom tomatoes are often characterized as being highly pigmented, and their variety names frequently reflect this with descriptors such as “purple”, “black”, or “zebra” [22,27]. Varieties such as Black Krim, Cherokee Purple, Brandywine, and Green Zebra exhibit a wide range of cultivar-dependent pigmentation, which likely stems from antioxidants of the flavonoid class. Such compounds have been found in high abundance in other types of crimson, red, and purple pigmented fruits, and may also be found in highly pigmented tomato varieties [28]. In correlation, heirloom tomatoes have shown an increased antioxidant capacity compared with commercial varieties [29]. As a result, these tomato varieties may be more attractive to the consumer, not only because of the current local and authentic food trend, but also because of the presumed increased health benefits of consuming a diet rich in antioxidants [27].

While heirloom tomatoes are popular and there exists significant consumer demand, most cultivars exhibit low disease resistance and decreased productivity compared with commercial standards [30,31,32,33,34,35]. Soil cultivation is directly linked to plant pathogenic bacteria and fungi [36,37]; thus, hydroponic cultivation removes the influence of soil-borne disease from the production cycle and has been shown to reduce disease prevalence and increase yield [34,38]. Additionally, hydroponic systems operated in controlled environments allow for year-round commercial production, which may enable local production as is desired by consumers [39,40]. Additionally, grafting onto productive (generative) rootstocks may provide an advantage in regard to the control of pathogens, season extension, and improved productivity [22]. In fact, the prevalence of two common infectious diseases of tomatoes, bacterial wilt (*Ralstonia solanacearum*) and fusarium wilt (*Fusarium oxysporum* Schltdl.:Fr. f. sp. *lycopersici* (*Sacc*.)) [41,42], has been significantly reduced by grafting onto disease resistant rootstocks [30,31,32,33,34,35]. However, while some research projects have shown no effects of grafting on antioxidant capacity of tomatoes [22,43,44,45,46], others have shown a correlation between grafting and a decrease in antioxidant levels [45]. Nevertheless, none of these studies have investigated hydroponic systems; therefore, little is known about the comparative effects of grafting on the antioxidative properties of standard and heirloom tomatoes in hydroponic cultivation.

## 2. Results

### 2.1. Maturity of Tomato Samples

**Figure 1:**
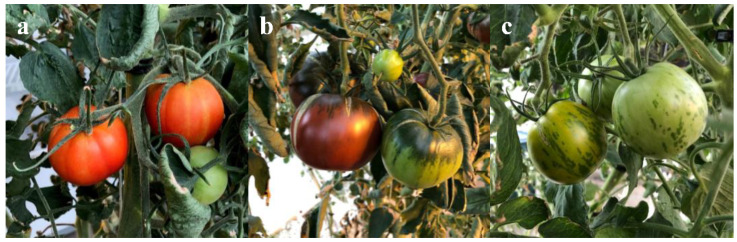
Standard (Big beef) and heirloom (Black Krim and Green Zebra) scions were grafted onto WT (self-graft), Arnold, or Supernatural rootstocks. Plants were grown in hydroponic culture at the Western Illinois University School of Agriculture Greenhouse facility, Macomb, IL. (**a**) Big Beef. (**b**) Black Krim. (**c**) Green Zebra.

Standard (Big Beef), and heirloom (Black Krim and Green Zebra; Figure 1) tomato varieties were grafted onto Arnold or Supernatural, or they were WT rootstock self-grafts. The percent soluble solids were not statistically significantly different, indicating that the tomatoes were harvested at approximately equal maturity (4–7% soluble solids) as determined by a Brix refractometer (Table 1).

### 2.2. Lycopene Content

Hexane extracts of lycopene were made from freeze-dried tomato powders. The lycopene content was determined spectrophotometrically at 503 nm. As expected, based on the natural pigmentation of each variety, the results for self-grafted tomatoes showed that the lycopene content of Black Krim was significantly higher, compared with Big Beef (*p* < 0.05). Green Zebra exhibited significantly less lycopene, compared with both Big Beef and Black Krim (*p* < 0.05), which likely reflects the lack of red pigmentation in the former.

When comparing the effect of grafting onto non-WT rootstocks, Big Beef grafted onto Arnold and Supernatural, and Green Zebra grafted onto Supernatural, demonstrated a correlation coefficient, r, above 0.7, indicating a strong correlation. Nevertheless, the correlation was not statistically significant (*p* > 0.05; Table 2).

### 2.3. Antioxidant Capacity

Samples for the determination of antioxidant capacity was made by extracting freeze-dried tomato powders with 1% HCl in 90% aqueous methanol. For the ABTS assay, ABTS^•^ was incubated with 10 μL tomato extract, after which the absorbance at 734 nm was measured. Trolox was used as an antioxidant standard to calculate TE (μmol/g tomato dry weight). The results showed that neither the commercial Big Beef nor the heirloom Black Krim or Green Zebra cultivars showed a correlation between the antioxidant capacity and the type of rootstock used for grafting (Table 3). These results indicate that the type of rootstock does not influence the antioxidant capacity under hydroponic cultivation as determined by the ABTS assay.

The DPPH assay was conducted by incubating tomato extracts with 10 μM DPPH, for 15 min, in the dark. Antioxidants reduce the absorbance of DPPH at 517 nm; thus, the starting absorbance was measured before the addition of tomato extracts. The starting absorbance at 517 nm was consistently between 0.510 and 0.540. After the addition of extracts, the decrease in absorbance was measured, and the trolox was used as a standard to calculate TE. The results showed that there is a strong correlation between the antioxidant capacities of Big Beef and Green Zebra grafted onto Supernatural, compared with the self-grafted cultivars, respectively (Table 4).

### 2.4. Phenolic Content

Total phenolic content was determined by the Folin–Ciocalteu assay, for which gallic acid was used as a standard for calculations of GAE. The phenolic content of Big Beef grafted onto both Arnold and Supernatural was strongly correlated, compared with self-grafted Big Beef. Further, the results showed that there is a strong correlation between the phenolic content of Green Zebra grafted onto Arnold, compared with the self-grafted cultivar (Table 5). These results indicate that the rootstock variety may influence the total phenolic content as well as the antioxidant capacity of certain standard and heirloom tomato varieties.

## 3. Discussion

Consumers are increasingly interested in produce that is grown locally and year-round and contains significant nutritional value [2,3,4]. Likewise, foods with a high content of antioxidants are receiving increased attention from consumers, due to the well-established relationship between these compounds and a reduced risk of cancer and cardiovascular diseases [47]. Studies have shown that tomatoes contain many types of antioxidants, including lycopene, flavonoids, and hydroxycinnamic acids, as well as other phenols [48,49,50]. However, while heirloom tomatoes are popular among consumers, the varieties are often challenging to produce due to disease susceptibility and low yield [30,32,33,34,38].

Hydroponic cultivation of commercial tomato varieties is known to both reduce disease susceptibility and to increase yield [13]. However, WT rootstocks of heirloom tomatoes often limit growth, resulting in reduced yield [22]. Although grafting onto productive and disease-resistant rootstock may provide increased growth and higher yield, little is known about the effects of these production and cultivation methods on the antioxidative properties of heirloom tomatoes. As consumers are becoming more aware of the health properties of food [2,3,4], the antioxidant capacity of heirloom tomatoes also becomes relevant to producers.

To investigate the effect of grafting on antioxidant capacity, standard Big Beef, heirloom Black Krim, and Green Zebra varieties were grown and then grafted onto Arnold and Supernatural rootstocks and then cultivated hydroponically. Harvested ripe tomatoes were freeze-dried, and extractions were made to analyze the lycopene and phenolic content, as well as the antioxidant capacity. The lycopene content varied between each variety of self-grafted tomatoes reflecting their natural pigmentation. The variation was evident from the significantly higher content of lycopene in Black Krim (dark purple), compared with Big Beef (red) and Green Zebra (green). Purple tomatoes have been shown to contain higher lycopene content compared with red varieties [51,52]. The low content of lycopene in Green Zebra has been previously reported [53] and reflects the lack of red pigmentation in this variety. In fact, it is well established that the typical red color of tomatoes is caused by lycopene [7,8]. Thus, the lycopene content of green tomatoes, such as Green Zebra or unripe fruits, is often non-detectable or significantly lower, compared with ripe and red varieties [53,54]. However, Pearson correlation analysis revealed a lack of correlation between the lycopene content of disease-resistant and WT rootstocks for all three tomato varieties. This observation corresponds to previous studies that have shown that the lycopene content remained unchanged [33,43]. However, others have reported a decrease in lycopene content as a result of grafting [45,55]. The differences in lycopene, as observed in the previous studies, may be related to using other combinations of rootstock and tomato varieties. The previous studies used Florida 47 grafted onto Beaufort and Multifort [33] and Tamaris grafted onto Efialto, Herman, and Maxifort rootstocks [45]. To our knowledge, the effect of grafting of Big Beef, Black Krim, and Green Zebra onto Arnold and Supernatural on the lycopene content has not previously been reported. Furthermore, both studies [33,45] employed soil cultivation, in contrast to our study, which utilized hydroponic culture. Thus, a direct comparison of the results is complicated. However, Ajlouni et al. compared the effect of soil and hydroponic cultures on the lycopene content of tomatoes (Pyramid) and found no significant difference [56]. Others have shown comparable outcomes, in which grafting of heirloom tomatoes improved fruit yield, but failed to influence fruit chemistry. However, these studies were carried out on tomatoes in soil culture [22,43].

Other research groups have provided evidence that hydroponic culture increases the overall antioxidant capacity of vegetables and herbs [57,58]. However, to our knowledge, the effect of grafting on the antioxidant capacity of hydroponic tomatoes has not previously been investigated. Here, the effect of grafting of tomatoes in hydroponic culture on the antioxidant capacity was further explored using ABTS and DPPH assays. The results from the ABTS assay showed no correlation between the antioxidant capacities for any of the tomato varieties and rootstock combinations. Nonetheless, a positive correlation between grafting onto Supernatural of both Big Beef and Green Zebra was observed on the antioxidant capacity, using the DPPH assay. While some studies have shown a decrease in the antioxidant capacity of grafted compared with non-grafted tomatoes [45], others have found no significant effects [22,43]. However, to our knowledge, no changes in the total antioxidant levels have been reported when comparing self-grafting to non-wild type rootstocks [44]. For example, Barrett et al. and Soare et al. showed that the antioxidant capacity, as determined by the DPPH assay, and the levels of the antioxidant ascorbic acid remained unchanged for self-grafted, compared with hybrid rootstocks [22,44]. Thus, our results may be the first to report an increase in antioxidant activity when grafting onto non-WT rootstocks.

When comparing our results from the ABTS and DPPH assays, an overarching increase for antioxidant capacity is seen in the ABTS results, compared with the DPPH. The general higher levels of antioxidant capacity for the ABTS are supported by previous work in comparing these two assays [59]. The ABTS assay has been found to report higher levels in comparison to the DPPH assay, when reacting with oxygen radicals, hydrophilic compounds, and highly pigmented compounds [59,60]. Solvent and reaction kinetic studies have shown that DPPH in methanol results in lower measured antioxidant capacity values compared with water [61]. This effect is explained by the hydrogen atom transfer (HAT) mechanism, which is reduced in strong hydrogen-bond accepting solvents, such as methanol [62]. Moreover, the increase in the antioxidant capacity of Big Beef and Green Zebra grafted onto Supernatural, as determined by the DPPH assay, was not reflected in the measured lycopene content. Although lycopene is a potent antioxidant, its high lipophilicity prevents its extraction by utilizing hydrophilic methods, as those used for the ABTS and DPPH assays [63,64].

Finally, the phenolic content of each tomato cultivar was determined by the Folin–Ciocalteu assay. A correlation for the phenolic content between Big Beef grafted onto Arnold and Supernatural, and Green Zebra grafted onto Arnold, was observed. Interestingly, the phenolic content increased for the Big Beef cultivars, whereas grafting of Green Zebra onto Arnold resulted in a decrease in the phenolic content. A similar correlation was found by Soare et al., who showed that the grafting of Lorely F1 onto Beaufort rootstock resulted in a significant decrease in the phenolic content [44]. Additionally, antioxidant levels in tomatoes depend not only on the cultivar and culture conditions but also on the maturity level of the fruit [65]. In fact, previous investigations have demonstrated a positive correlation between maturity level and phenolic content [66,67,68,69]. In this study, Green Zebra grafted onto Arnold rootstock exhibited the lowest soluble solid percentage, an indicator of fruit maturity, of the Green Zebra cultivars. However, this difference was not found to be statistically significantly.

The observed changes in antioxidant and phenolic content indicate that tomato crop management may influence the biosynthesis of secondary metabolites. Several studies have investigated the effects of grafting on secondary metabolites, including phenolic compounds. These studies have shown that grafting generally increases flavonoid content while decreasing other secondary metabolites such as theanine and caffeine [70,71,72,73,74,75]. However, studies on the comparative effects of different grafting rootstocks on the biosynthesis of secondary metabolites are limited. Zombardo et al. showed that, at maturity, genes were differentially expressed in the berries of grapevine (*Vitis vinifera* L.) grafted onto Paulsen and Mgt 101–14 rootstocks and that these genes were involved in the synthesis and transport of flavonoids. These data were further supported by the profiling of the phenolic content, which showed lower levels in Mgt 101–14, compared with Paulsen rootstocks [76]. Thus, in this study, the observed changes in lycopene and phenolic content as a result of grafting onto different rootstocks may reflect the differential regulation of genes responsible for biosynthesis of secondary metabolites. However, these results may not be observed in other generalized forms and must be validated in future studies.

## 4. Materials and Methods

### 4.1. Materials

6-hydroxy-2,5,7,8-tetramethylchroman-2-carboxylic (trolox), 2,2′-azinobis-(3-ethylbenzothiazoline-6-sulfonate) (ABTS), 2,2-diphenyl-1-picryl-hydrazyl-hydrate (DPPH), and 2 N Folin–Ciocalteu reagent were purchased from Sigma Aldrich (St. Louis, MO, USA). Potassium persulfate was purchased from MCM chemicals (Cleveland, OH, USA), and gallic acid from Millipore Chemicals (St. Louis, MO, USA). All other chemicals and reagents were purchased from Fischer Scientific (Hampton, NH, USA).

### 4.2. Plants

Scions of two heirloom (Black Krim and Green Zebra), one commercial standard (Big Beef), and rootstocks (wild-type; WT, Arnold or Supernatural) were produced by planting one seed per cell into 72-cell trays filled with peat-based growing mix (ProMix BS, with Biofungicide, Premier Tech Horticulture, Cromwell, MN, USA), on 12 December 2017, at the Western Illinois University School of Agriculture Greenhouse facility, Macomb, IL. The plants were transported to the laboratory, on 15 January 2018, prior to being splice-grafted, as previously described [77]. In brief, the rootstock and scion stems were cut at a deep angle above the cotyledons, and the cut surfaces of the rootstock and scion were held together by a grafting clip. The grafted plants were maintained at 20 °C and 95% humidity by misting with a hand-held sprayer, as necessary. After 5 d, humidity was gradually reduced to ambient over 7 d, after which plants were reintroduced to the greenhouse. Three replicates of each scion/stock combination were prepared for evaluation.

The hydroculture system utilized for post-grafted growth and production was a containerized recirculating system. Two tomato plants were transplanted per 11 L hydroponic greenhouse pot (Bato troughs; Hort Americas, Bedford, TX, USA) containing coarse perlite (Deerfield Supplies, Elkton, KY, United States) for the remainder of the trial, on 29 January 2018. A two-part complete hydroponic fertilizer (CropKing; Lodi, OH, USA), consisting of a complete fertilizer (4.4 N-13.0 P-34.0 K; HydroGro Vine Crops, Scottsdale, AZ, United States), supplemented with greenhouse-grade calcium nitrate (15.5 N-0.0 P-0.0 K; Yara North America, Tampa, FL, USA) fertilizer was mixed, as per manufacturer instructions. The fertilizer solution was monitored daily and adjusted when necessary, to maintain at an electrical conductivity of 2000 µS cm^−1^ and a pH of 5.5 and was replaced at 14 d intervals. The plants were exposed to a 12:12 h light-dark cycle, and irrigation scheduling was set for 30 s every 30 m during the lighted portion of the growing cycle. Greenhouse temperature was maintained at 24 °C by thermostatic monitoring and automatic heating and cooling as necessary.

### 4.3. Maturity of Tomato Samples

The tomatoes were harvested when vine-ripe (100% red or equivalent), prior to transfer to the laboratory for further evaluation. Only US No. 1 grade tomatoes were used for laboratory analysis [77]. As a means to further ensure sample homogeneity, the harvested fruits were further separated via digital refractometer with only those exhibiting 4–7% soluble solids (Brix), used for laboratory analysis. Tomatoes chosen for evaluation were finely sliced, lyophilized, and ground into powder using a pestle and mortar at 4 °C. Tomato powders were stored at −20 °C until further analysis.

### 4.4. Determination of Lycopene Content

Freeze-dried tomato powder (100 mg) was extracted by 1 mL hexane/acetone/ethanol (2:1:1, *v*/*v*/*v*) under shaking for 30 min, at room temperature, after which 0.2 mL ddH2O was added and vigorously mixed. The polar and non-polar phases were separated by centrifugation at 3000× *g* for 10 min, after which the non-polar phase containing lycopene was collected. The remaining plant material was extracted once more using 1 mL hexane/acetone/ethanol (2:1:1, *v*/*v*/*v*), as described above. The content of lycopene was determined by measuring the absorbance spectrophotometrically at 503 nm (εlycopene = 1.72 × 105 M^−1^ cm^−1^).

### 4.5. Antioxidant Capacity

The samples (100 mg) were extracted with aqueous 90% methanol with 1% (*v*/*v*) HCl, for 2 h under shaking, at room temperature, as described by others [78]. The samples were centrifuged for 90 s at 1000× *g*, and the supernatant was collected and stored at −20 °C until further use.

For the 2,2′-azino-bis(3-ethylbenzothiazoline-6-sulphonic acid) (ABTS) assay, the ABTS radical (ABTS•) was made by incubating equal volumes of 7 mM ABTS and 2.45 mM potassium persulfate for 12–18 h in the dark. To measure the antioxidant capacity, 10 L tomato extract was incubated with 95 L 3.5 mM ABTS• for 30 s at room temperature, and the absorbance at 734 nm was measured spectrophotometrically (Spectra Max 250 Microplate Reader, Molecular Devices, San Jose, CA, USA). Trolox was used as an antioxidant standard for calculations of trolox equivalents (TE; μmol/g tomato dry weight).

Prior to each 2,2-diphenyl-1-picrylhydrazyl (DPPH) assay, 10 μM DPPH in 90% aqueous methanol was measured spectrophotometrically at 517 nm (Spectra Max 250 Microplate Reader, Molecular Devices, San Jose, CA, USA) to ensure that the absorbance was between 0.510 and 0.540. To determine the antioxidant capacity, 10 L tomato extract was mixed with 195 µL 10 μM DPPH in 90% aqueous methanol and incubated in the dark for 15 min. The decrease in absorbance at 517 nm was then measured. Trolox was used as an antioxidant standard for calculations of TE.

### 4.6. Phenolic Content

Total phenolic content was determined by the Folin–Ciocalteu assay. In brief, 20 L tomato extract was incubated with 10 L 2 N Folin–Ciocalteu reagent, 100 L ddH2O, and 120 L 12.5% sodium carbonate, and incubated for 30 min. Phenolic content was then measured spectrophotometrically at 750 nm (Spectra Max 250 Microplate Reader, Molecular Devices, San Jose, CA, USA). Gallic acid was used as a phenolic standard for calculations of gallic acid equivalents (GAE; μmol/g tomato dry weight).

### 4.7. Statistical Analysis

A Pearson correlation was performed between all sets of data. The data were considered as correlated when the correlation coefficient (r) was < −0.7 (negative correlation) or r > 0.7 (positive correlation). A one-way ANOVA was performed to evaluate statistical significance. A *p*-value of <0.05 was considered significant. Statistical analysis was performed using Grahpad Prism (v.s. 9.1.0; San Diego, CA, USA).

## 5. Conclusions

In conclusion, a correlation between the grafting of standard (Big Beef) and heirloom (Black Krim and Green Zebra) onto Arnold and Supernatural rootstocks, compared with self-grafted cultivars was observed for the antioxidant capacity and phenolic content, while the percent soluble solids and lycopene content were not correlated. Specifically, a positive correlation was observed for the antioxidant capacity, as determined by DPPH, for Big Beef and Green Zebra grafted onto Supernatural. Interestingly, this effect was not measured by the ABTS assay, most likely reflecting the differences in the molecular mechanisms of the two antioxidant capacity assays. Furthermore, a correlation between grafting and the tomato variety was observed for the phenolic content. In particular, Big Beef grafted onto Arnold and Supernatural, and Green Zebra grafted onto Arnold, were correlated, indicating that the rootstock variety influences this aspect of fruit chemistry. These results are interesting and may provide valuable information to producers when choosing the most optimal combination of tomato and rootstock varieties.

## Figures and Tables

**Table 1:** The soluble solid content of tomato samples (mean ± std).

Tomato Sample (Variety/Rootstock)	Soluble Solids (%)
Big Beef/WT	5.92 ± 0.63
Big Beef/Arnold	5.20 ± 0.53
Big Beef/Supernatural	5.50 ± 0.24
Black Krim/WT	6.30 ± 0.68
Black Krim/Arnold	6.05 ± 0.71
Black Krim/Supernatural	5.30 ± 0.69
Green Zebra/WT	6.95 ± 0.71
Green Zebra/Arnold	5.60 ± 0.00
Green Zebra/Supernatural	5.75 ± 0.92

**Table 2:** Lycopene content (mg/kg dry weight) of tomato extracts. The lycopene content was significantly different for self-grafted Big Beef, Black Krim, and Green Zebra. Different letters (a, b, c) indicate statistical significance. A *p*-value of <0.05 was considered significant. Analysis using Pearson correlation showed a strong correlation between Big Beef grafted onto Arnold and Supernatural and Green Zebra grafted onto Supernatural, compared with their respective self-grafted cultivars. However, the correlation was not significant. The correlation coefficient of r < −0.7 or r > 0.7 was considered a strong correlation.

Tomato Cultivar (Variety/Rootstock)	Lycopene (Mean ± std)	Correlation Coefficient r
Big Beef/WT	9.97 *±* 2.02 ^a^	−
Big Beef/Arnold	8.09 *±* 1.39 ^a^	0.990
Big Beef/Supernatural	17.25 *±* 0.24 ^b^	0.986
Black Krim/WT	15.55 *±* 1.97 ^b^	−
Black Krim/Arnold	16.29 *±* 2.93 ^b^	0.083
Black Krim/Supernatural	19.14 *±* 3.21 ^b^	0.509
Green Zebra/WT	3.37 *±* 0.39 ^c^	−
Green Zebra/Arnold	3.52 *±* 0.15 ^c^	0.295
Green Zebra/Supernatural	3.03 *±* 0.03 ^c^	−0.899

**Table 3:** Antioxidant capacity of tomato extracts as analyzed by the ABTS assay. Antioxidant capacity was calculated as trolox equivalents (TE; μmol/g tomato dry weight). Analysis using Pearson correlation showed that the antioxidant capacity was not strongly correlated. A correlation coefficient of r < −0.7 or r > 0.7 was considered a strong correlation.

Tomato Cultivar (Variety/Rootstock)	TE (Mean ± std)	Correlation Coefficient r
Big Beef/WT	12.18 *±* 0.82	−
Big Beef/Arnold	12.34 *±* 0.65	0.406
Big Beef/Supernatural	12.26 *±* 0.79	0.025
Black Krim/WT	12.35 *±* 0.83	−
Black Krim/Arnold	11.99 *±* 1.31	−0.031
Black Krim/Supernatural	11.60 *±* 1.54	0.234
Green Zebra/WT	11.42 *±* 1.12	−
Green Zebra/Arnold	11.76 *±* 1.93	−0.320
Green Zebra/Supernatural	11.71 *±* 0.83	−0.014

**Table 4:** Antioxidant capacity of tomato extracts as analyzed by the DPPH assay. Antioxidant capacity was calculated as trolox equivalents (TE; μmol/g tomato dry weight). Analysis using Pearson correlation showed that the antioxidant capacity was strongly correlated for Big Beef grafted onto Supernatural. A correlation coefficient of r < −0.7 or r > 0.7 was considered a strong correlation.

Tomato Cultivar (Variety/Rootstock)	TE (Mean ± std)	Correlation Coefficient r
Big Beef/WT	7.88 ± 0.72	−
Big Beef/Arnold	7.62 ± 0.87	0.529
Big Beef/Supernatural	7.99 ± 0.68	0.826 **
Black Krim/WT	7.59 ± 0.78	−
Black Krim/Arnold	8.11 ± 0.54	0.080
Black Krim/Supernatural	7.70 ± 0.88	0.300
Green Zebra/WT	8.00 ± 0.53	−
Green Zebra/Arnold	7.66 ± 0.78	0.638
Green Zebra/Supernatural	8.19 ± 0.64	0.783 *

* Correlation is significant at the *p* < 0.05 level (two-tailed). ** Correlation is significant at the *p* < 0.01 level (two-tailed).

**Table 5:** Total phenolic content of tomato extracts as analyzed by the Folin–Ciocalteu assay. Phenolic content was calculated as gallic acid equivalents (GAE; μmol/g tomato dry weight). Analysis using Pearson correlation showed that the antioxidant capacity was strongly correlated for Big Beef grafted onto Arnold and Supernatural and Green Zebra grafted onto Arnold, compared with the self-grafted cultivars. A correlation coefficient of r < −0.7 or r > 0.7 was considered a strong correlation.

Tomato Cultivar (Variety/Rootstock)	GAE (Mean ± std)	Correlation Coefficient r
Big Beef/WT	13.57 ± 3.14	−
Big Beef/Arnold	14.34 ± 2.40	0.927 ***
Big Beef/Supernatural	15.80 ± 2.77	0.831 **
Black Krim/WT	14.40 ± 2.72	−
Black Krim/Arnold	15.29 ± 2.93	0.164
Black Krim/Supernatural	13.59 ± 2.58	0.087
Green Zebra/WT	16.97 ± 4.03	−
Green Zebra/Arnold	10.95 ± 4.22	0.989 ****
Green Zebra/Supernatural	15.84 ± 2.81	0.649

** Correlation is significant at the *p* < 0.01 level (two-tailed). *** Correlation is significant at the *p* < 0.001 level (two-tailed). **** Correlation is significant at the *p* < 0.0001 level (two-tailed).

## Data Availability

The data from this research is available from Mendeley Data (http://dx.doi.org/10.17632/8g6x6frsdr.3, http://dx.doi.org/10.17632/88z4g43b84.2, http://dx.doi.org/10.17632/w3m8wr6344.1). Accessed on 11 May 2021.

## References

[1] Joseph H., Nink E., McCarthy A., Messer E., Cash S.B. (2017). The Heirloom Tomato is ‘In’. Does It Matter How It Tastes?. Food Cult. Soc..

[2] Bland S.E. (2005). Consumer Acceptability of Heirloom Tomatoes.

[3] Loureiro M.L., Hine S. (2015). Discovering Niche Markets: A Comparison of Consumer Willingness to Pay for Local (Colorado Grown), Organic, and GMO-Free Products. J. Agric. Appl. Econ..

[4] Reimers K.J.P., Keast D.R.P. (2016). Tomato Consumption in the United States and Its Relationship to the US Department of Agriculture Food Pattern: Results From What We Eat in America 2005–2010. Nutr. Today.

[5] Aghajanpour M., Nazer M.R., Obeidavi Z., Akbari M., Ezati P., Kor N.M. (2017). Functional foods and their role in cancer prevention and health promotion: A comprehensive review. Am. J. Cancer Res..

[6] Chang S.K., Alasalvar C., Shahidi F. (2019). Superfruits: Phytochemicals, antioxidant efficacies, and health effects—A comprehensive review. Crit. Rev. Food Sci. Nutr..

[7] Imran M., Ghorat F., Ul-Haq I., Ur-Rehman H., Aslam F., Heydari M., Shariati M.A., Okuskhanova E., Yessimbekov Z., Thiruvengadam M. (2020). Lycopene as a Natural Antioxidant Used to Prevent Human Health Disorders. Antioxidants.

[8] Li N., Wu X., Zhuang W., Xia L., Chen Y., Wu C., Rao Z., Du L., Zhao R., Yi M. (2021). Tomato and lycopene and multiple health outcomes: Umbrella review. Food Chem..

[9] Ruskovska T., Maksimova V., Milenkovic D. (2020). Polyphenols in human nutrition: From the in vitro antioxidant capacity to the beneficial effects on cardiometabolic health and related inter-individual variability—An overview and perspective. Br. J. Nutr..

[10] Benzie I.F.F., Choi S.-W. (2014). Antioxidants in food: Content, measurement, significance, action, cautions, caveats, and research needs. Adv. Food Nutr. Res..

[11] Rahal A., Kumar A., Singh V., Yadav B., Tiwari R., Chakraborty S., Dhama K. (2014). Oxidative stress, prooxidants, and antioxidants: The interplay. BioMed Res. Int..

[12] Schieber M., Chandel N.S. (2014). ROS Function in Redox Signaling and Oxidative Stress. Curr. Biol..

[13] Figueroa-Mendez R., Rivas-Arancibia S. (2015). Vitamin C in Health and Disease: Its Role in the Metabolism of Cells and Redox State in the Brain. Front. Physiol..

[14] Mittler R. (2017). ROS Are Good. Trends Plant Sci..

[15] Nissanka N., Moraes C.T. (2018). Mitochondrial DNA damage and reactive oxygen species in neurodegenerative disease. FEBS Lett..

[16] Huang Z., Chen Y., Zhang Y. (2020). Mitochondrial reactive oxygen species cause major oxidative mitochondrial DNA damages and repair pathways. J. Biosci..

[17] Cadet J., Wagner J.R. (2013). DNA Base Damage by Reactive Oxygen Species, Oxidizing Agents, and UV Radiation. Cold Spring Harb. Perspect. Biol..

[18] Zhang Z., Weichenthal S., Kwong J.C., Burnett R.T., Hatzopoulou M., Jerrett M., van Donkelaar A., Bai L., Martin R.V., Copes R. (2021). A Population-Based Cohort Study of Respiratory Disease and Long-Term Exposure to Iron and Copper in Fine Particulate Air Pollution and Their Combined Impact on Reactive Oxygen Species Generation in Human Lungs. Environ. Sci. Technol..

[19] Matic I. (2018). The major contribution of the DNA damage-triggered reactive oxygen species production to cell death: Implications for antimicrobial and cancer therapy. Curr. Genet..

[20] Klein D., Gkisakis V., Krumbein A., Livieratos I., Kopke U. (2010). Old and endangered tomato cultivars under organic greenhouse production: Effect of harvest time on flavour profile and consumer acceptance. Int. J. Food Sci. Technol..

[21] Vela-Hinojosa C., Escalona-Buendía H.B., Mendoza-Espinoza J.A. (2019). Antioxidant Balance and Regulation in Tomato Genotypes of Different Color. J. Am. Soc. Hortic. Sci..

[22] Barrett C.E., Zhao X., Sims C.A., Brecht J.K., Dreyer E.Q., Gao Z.F. (2012). Fruit Composition and Sensory Attributes of Organic Heirloom Tomatoes as Affected by Grafting. Horttechnology.

[23] Zivanovic B., Vidovic M., Milic Komic S., Jovanovic L., Kolarz P., Morina F., Veljovic Jovanovic S. (2017). Contents of phenolics and carotenoids in tomato grown under polytunnels with different UV-transmission rates. Turk. J. Agric. For..

[24] Casals J., Rull A., Bernal M., Gonzalez R., Romero del Castillo R., Simo J. (2018). Impact of grafting on sensory profile of tomato landraces in conventional and organic management systems. Hortic. Environ. Biotechnol..

[25] D’Angelo M., Zanor M.I., Sance M., Cortina P.R., Boggio S.B., Asprelli P., Carrari F., Santiago A.N., Asis R., Peralta I.E. (2018). Contrasting metabolic profiles of tasty Andean varieties of tomato fruit in comparison with commercial ones. J. Sci. Food Agric..

[26] Fraser G.E., Jacobsen B.K., Knutsen S.F., Mashchak A., Lloren J.I. (2020). Tomato consumption and intake of lycopene as predictors of the incidence of prostate cancer: The Adventist Health Study-2. Cancer Cause Control.

[27] Wang D., Seymour G.B. (2017). Tomato Flavor: Lost and Found?. Mol. Plant.

[28] Kang S.-I., Rahim M.A., Afrin K.S., Jung H.-J., Kim H.-T., Park J.-I., Nou I.-S. (2018). Expression of anthocyanin biosynthesis-related genes reflects the peel color in purple tomato. Hortic. Environ. Biotechnol..

[29] Alonso A., Salazar J.A., Arroyo A., Grau A., Garcia-Martinez S., Serrano M., Ruiz J.J. (2011). Screening a diverse collection of heirloom tomato cultivars for quality and functional attributes. Acta Hortic..

[30] Di Gioia F., Serio F., Buttaro D., Ayala O., Santamaria P. (2010). Influence of rootstock on vegetative growth, fruit yield and quality in ’Cuore di Bue’, an heirloom tomato. J. Hortic. Sci. Biotechnol..

[31] Rivard C.L. (2008). Grafting to Manage Soilborne Diseases in Heirloom Tomato Production. Hortsceince.

[32] Frey C.J., Zhao X., Brecht J.K., Huff D.M., Black Z.E. (2020). High Tunnel and Grafting Effects on Organic Tomato Plant Disease Severity and Root-knot Nematode Infestation in a Subtropical Climate with Sandy Soils. Hortscience.

[33] Lang K.M., Nair A. (2019). Effect of Tomato Rootstock on Hybrid and Heirloom Tomato Performance in a Midwest High Tunnel Production System. Hortscience.

[34] Lang K.M., Nair A. (2018). In the Absence of Soil-borne Disease Pressure, Does Tomato Grafting Still Benefit Midwest Vegetable Growers?. Hortscience.

[35] Suchoff D.H., Louws F.J., Schultheis J.R., Kleinhenz M.D., Gunter C.C. (2017). Rootstock-imparted Water Use Efficiency in Grafted Heirloom Tomatoes. Hortscience.

[36] Weller D.M., Raaijmakers J.M., Gardener B.B.M., Thomashow L.S. (2002). Microbial populations responsible for specific soil suppressiveness to plant pathogens. Annu. Rev. Phytopathol..

[37] van Agtmaal M., Straathof A.L., Termorshuizen A., Lievens B., Hoffland E., de Boer W. (2018). Volatile-mediated suppression of plant pathogens is related to soil properties and microbial community composition. Soil Biol. Biochem..

[38] Testen A.L., Bosques Martinez M., Jimenez Madrid A., Deblais L., Taylor C., Paul P.A.D., Miller S.A. (2020). On-farm evaluations of anaerobic soil disinfestation and grafting for management of a widespread soilborne disease complex in protected culture tomato production. Phytopathology.

[39] Barbosa Guilherme L., Gadelha Francisca Daiane A., Kublik N., Proctor A., Reichelm L., Weissinger E., Wohlleb Gregory M., Halden Rolf U. (2015). Comparison of Land, Water, and Energy Requirements of Lettuce Grown Using Hydroponic vs. Conventional Agricultural Methods. Int. J. Environ. Res. Public Health.

[40] Goddek S., Vermeulen T. (2018). Comparison of Lactuca sativa growth performance in conventional and RAS-based hydroponic systems. Aquac. Int..

[41] Snyder W.C., Hansen H.N. (1940). The Species Concept in Fusarium. Am. J. Bot..

[42] Yabuuchi E., Kosako Y., Oyaizu H., Yano I., Hotta H., Hashimoto Y., Ezaki T., Arakawa M. (1992). Proposal of Burkholderia gen. nov. and transfer of seven species of the genus Pseudomonas homology group II to the new genus, with the type species Burkholderia cepacia (Palleroni and Holmes 1981) comb. nov. Microbiol. Immunol..

[43] Djidonou D., Simonne A.H., Koch K.E., Brecht J.K., Zhao X. (2016). Nutritional Quality of Field-grown Tomato Fruit as Affected by Grafting with Interspecific Hybrid Rootstocks. Hortscience.

[44] Soare R., Dinu M., Babeanu C. (2018). The effect of using grafted seedlings on the yield and quality of tomatoes grown in greenhouses. Hortic. Sci..

[45] Vrcek V. (2011). The effect of grafting on the antioxidant properties of tomato *(Solanum lycopersicum* L.). Span. J. Agric. Res..

[46] Arias Padro M.D., Caboni E., Salazar Morin K.A., Meraz Mercado M.A., Olalde-Portugal V. (2021). Effect of Bacillus subtilis on antioxidant enzyme activities in tomato grafting. PeerJ.

[47] Serafini M., Peluso I. (2016). Functional Foods for Health: The Interrelated Antioxidant and Anti-Inflammatory Role of Fruits, Vegetables, Herbs, Spices and Cocoa in Humans. Curr. Pharm. Des..

[48] Blando F., Berland H., Maiorano G., Durante M., Mazzucato A., Picarella M.E., Nicoletti I., Gerardi C., Mita G., Andersen O.M. (2019). Nutraceutical Characterization of Anthocyanin-Rich Fruits Produced by “Sun Black” Tomato Line. Front. Nutr..

[49] Blando F., Calabriso N., Berland H., Maiorano G., Gerardi C., Carluccio M.A., Andersen O.M. (2018). Radical Scavenging and Anti-Inflammatory Activities of Representative Anthocyanin Groupings from Pigment-Rich Fruits and Vegetables. Int. J. Mol. Sci..

[50] Jordan J.A. (2007). The Heirloom Tomato as Cultural Object: Investigating Taste and Space. Sociol. Rural..

[51] Campestrini L.H., Melo P.S., Peres L.E.P., Calhelha R.C., Ferreira I.C.F.R., Alencar S.M. (2019). A new variety of purple tomato as a rich source of bioactive carotenoids and its potential health benefits. Heliyon.

[52] Hazra P., Longjam M., Chattopadhyay A. (2018). Stacking of mutant genes in the development of "purple tomato" rich in both lycopene and anthocyanin contents. Sci. Hortic..

[53] Kumar R., Klein D., Krumbein A., Kopke U. (2007). Product quality of greenhouse tomatoes: Effect of cultivars, organic N-fertilization and harvest time. Eur. J. Hortic. Sci..

[54] Badejo A.A., Adebowale A.P., Enujiugha V.N. (2016). Changes in Nutrient Composition, Antioxidant Properties, and Enzymes Activities of Snake Tomato (Trichosanthes cucumerina) during Ripening. Prev. Nutr. Food Sci..

[55] Helyes L., Lugasi A., Pogonyi A., Pek Z. (2009). Effect of variety and grafting on lycopene content of tomato (lycopersicon lycopersicum l. karsten) fruit. Acta Aliment..

[56] Ajlouni S.K., Masih L. (2001). Lycopene content in hydroponic and non-hydroponic tomatoes during postharvest storage. Food Aust..

[57] Kim J.S., An C.G., Park J.S., Lim Y.P., Kim S. (2016). Carotenoid profiling from 27 types of paprika (*Capsicum annuum* L.) with different colors, shapes, and cultivation methods. Food Chem..

[58] Sgherri C., Cecconami S., Pinzino C., Navari-Izzo F., Izzo R. (2010). Levels of antioxidants and nutraceuticals in basil grown in hydroponics and soil. Food Chem..

[59] Gaber N.B., El-Dahy S.I., Shalaby E.A. (2021). Comparison of ABTS, DPPH, permanganate, and methylene blue assays for determining antioxidant potential of successive extracts from pomegranate and guava residues. Biomass Convers. Biorefinery.

[60] Bibi Sadeer N., Montesano D., Albrizio S., Zengin G., Mahomoodally M.F. (2020). The Versatility of Antioxidant Assays in Food Science and Safety-Chemistry, Applications, Strengths, and Limitations. Antioxidants.

[61] Abramovič H., Grobin B., Poklar Ulrih N., Cigić B. (2017). The methodology applied in DPPH, ABTS and Folin-Ciocalteau assays has a large influence on the determined antioxidant potential. Acta Chim. Slov..

[62] Huang D., Ou B., Prior R.L. (2005). The Chemistry behind Antioxidant Capacity Assays. J. Agric. Food Chem..

[63] Prior R.L., Wu X., Schaich K. (2005). Standardized Methods for the Determination of Antioxidant Capacity and Phenolics in Foods and Dietary Supplements. J. Agric. Food Chem..

[64] Yeo J., Shahidi F. (2019). Critical Re-Evaluation of DPPH assay: Presence of Pigments Affects the Results. J. Agric. Food Chem..

[65] Dinu M., Dumitru M.G., Soare R. (2015). The effect of some biofertilizers on the biochemical components of the tomato plants and fruits. Bulg. J. Agric. Sci..

[66] Al-Zyadi Q.A.S. (2019). Effect of nitrogen fertilization and harvesting date on growth of burdock plant (*Arctium lappa* L.) and total phenols content in leaves. Biochem. Cell. Arch..

[67] Liu C., Zheng H., Sheng K., Liu W., Zheng L. (2018). Effects of postharvest UV-C irradiation on phenolic acids, flavonoids, and key phenylpropanoid pathway genes in tomato fruit. Sci. Hortic..

[68] Slimestad R., Verheul M. (2009). Review of flavonoids and other phenolics from fruits of different tomato (*Lycopersicon esculentum* Mill.) cultivars. J. Sci. Food Agric..

[69] Tomas M., Beekwilder J., Hall R.D., Sagdic O., Boyacioglu D., Capanoglu E. (2017). Industrial processing versus home processing of tomato sauce: Effects on phenolics, flavonoids and in vitro bioaccessibility of antioxidants. Food Chem..

[70] Deng W.-w., Fan Y.-b., Gu C.-c., Li D.-x., Wan X.-c. (2017). Changes in Morphological Characters and Secondary Metabolite Contents in Leaves of Grafting Seedlings with Camellia sinensis as Scions and C. oleifera as Stocks. J. Trop. Subtrop. Bot..

[71] Xu D., Yuan H., Tong Y., Zhao L., Qiu L., Guo W., Shen C., Liu H., Yan D., Zheng B. (2017). Comparative Proteomic Analysis of the Graft Unions in Hickory (*Carya cathayensis*) Provides Insights into Response Mechanisms to Grafting Process. Front. Plant Sci..

[72] Yuan H., Zhao L., Qiu L., Xu D., Tong Y., Guo W., Yang X., Shen C., Yan D., Zheng B. (2017). Transcriptome and hormonal analysis of grafting process by investigating the homeostasis of a series of metabolic pathways in Torreya grandis cv. Merrillii. Ind. Crop. Prod..

[73] Deng W.-W., Han J., Fan Y., Tai Y., Zhu B., Lu M., Wang R., Wan X., Zhang Z.-Z. (2018). Uncovering tea-specific secondary metabolism using transcriptomic and metabolomic analyses in grafts of Camellia sinensis and C. oleifera. Tree Genet. Genomes.

[74] Prodhomme D., Fonayet J.V., Hevin C., Franc C., Hilbert G., de Revel G., Richard T., Ollat N., Cookson S.J. (2019). Metabolite profiling during graft union formation reveals the reprogramming of primary metabolism and the induction of stilbene synthesis at the graft interface in grapevine. BMC Plant Biol..

[75] Zhang X.-y., Sun X.-z., Zhang S., Yang J.-h., Liu F.-f., Fan J. (2019). Comprehensive transcriptome analysis of grafting onto Artemisia scoparia W. to affect the aphid resistance of chrysanthemum (*Chrysanthemum morifolium* T.). BMC Genom..

[76] Zombardo A., Crosatti C., Bagnaresi P., Bassolino L., Reshef N., Puccioni S., Faccioli P., Tafuri A., Delledonne M., Fait A. (2020). Transcriptomic and biochemical investigations support the role of rootstock-scion interaction in grapevine berry quality. BMC Genom..

[77] Guan W., Hallett S. Techniques for Tomato Grafting. https://extension.purdue.edu/extmedia/HO/HO-260-W.pdf.

[78] Martinez-Valverde I., Periago M.J., Provan G., Chesson A. (2002). Phenolic compounds, lycopene and antioxidant activity in commercial varieties of tomato (Lycopersicum esculentum). J. Sci. Food Agric..

